# Bordetella Bronchiseptica in the Immunosuppressed Population – A Case Series and Review

**DOI:** 10.4084/MJHID.2014.031

**Published:** 2014-04-07

**Authors:** Abraham T. Yacoub, Mitsuya Katayama, JoAnn Tran, Ronit Zadikany, Manasa Kandula, John Greene

**Affiliations:** 1 H. Lee Moffitt Cancer Center and Research Institute, 12902 Magnolia Drive, Tampa, Florida 33612-9497.; 2 University of South Florida, Division of Infectious Diseases and International Medicine, 1 Tampa General Circle, G323 Tampa, FL 33606; 3 University of South Florida Morsani College of Medicine, 12901 Bruce B. Down Blvd, Tampa, Fl 33612-4742; 4 H. Lee Moffitt Cancer Center and Research Institute, University of South Florida College of Medicine, 12902 Magnolia Drive, Tampa, Florida 33612-9497

## Abstract

Organisms that are not known to cause serious infection in the immunocompetent population can, in fact, cause devastating illness in immunosuppressed neutropenic populations especially those who are undergoing hematopoietic stem cell transplantation (HSCT), and solid organ transplantation or a history of malignancy. One organism of interest isolated from immunosuppressed patients at our institution was *Bordetella bronchiseptica*. It is known to cause respiratory tract disease in the animal population which includes dogs, cats, and rabbits. This organism rarely causes serious infection in the immunocompetent population. However; in immunosuppressed patients, it can cause serious pulmonary disease. We present three cases of *B. bronchiseptica* pneumonia in patients with a history of malignancy.

## Introduction

*Bordetella bronchiseptica* is an aerobic, motile, gram-negative rod most commonly viewed as a commensal organism that inhabits the upper respiratory tract of various domestic and feral mammals. Such animals include guinea pigs, rats, mice, ferrets, horses, chicken, mice, primates and koala bears. The spectrum of illnesses caused by *B. bronchiseptica* in susceptible animals includes tracheobronchitis of rabbits and guinea pigs (snuffles) and dogs (kennel cough), as well as turbinate atrophy in swine.^1^–^3^

While *B. bronchiseptica* infection has been found in this broad range of different hosts, it has two closely related *Bordetella* species, *B. pertussis* and *B. parapertussis*, that naturally infect humans.^4^ Despite potentially frequent exposure to zoonotic sources of this opportunistic agent, human infections are rare. As of 2006 fifty-five cases of human infection have been reported.^5^–^7^ We present 3 cases and literature review *B. bronchiseptica* in the immunosuppressed population.

## Case Series

### Case One

A 43-yr-old female presented with a 2-day history of increasing swelling of the face and right upper extremity. In addition, she had one week history of fever, progressive shortness of breath, and cough with yellow sputum production. Four months prior to admission, she was diagnosed with superior vena cava syndrome and malignant thymoma. She was treated with concomitant radiation and chemotherapy followed by radical excision of the thymoma and reconstruction of the right subclavian vein with a Gortex graft. She was admitted to the hospital and diagnosed with superior vena cava syndrome due to thrombosis of the venous graft. An unsuccessful attempt was made to lyse the thrombus with urokinase. She had been an active smoker of one pack of cigarettes a day for 20 years. She did not own a pet and denied any contact with animals but she had a history of cat scratch disease. Laboratory results revealed an elevated WBC count of 21 cells/ml. Sputum culture obtained at admission produced moderate growth (2+) of *B. bronchiseptica* and C. albicans. In vitro testing indicated sensitivity to amikacin, gentamicin, tobramycin and piperacillin. Blood and urine cultures were negative. Chest X-ray demonstrated bilateral pleural effusions and lower lung infiltrates. Intravenous cefotaxime 1gm thrice daily was begun empirically along with 1 dose of intravenous tobramycin 90 mg. On the fourth day of hospitalization, the dyspnea worsened. Chest x-ray demonstrated a left greater than right pleural effusion. CT-guided thoracentesis of the left pleural effusion drained 1 liter of a transudate-like fluid which was negative for acid fast bacilli, bacteria, fungi, and malignant cells.

One week after admission, the patient developed severe respiratory distress. Cefotaxime was changed to intravenous piperacillin 3g every four hours and tobramycin 100mg every eight hours. Sputum cultures again grew C. albicans as well as 4+ *B. bronchiseptica* sensitive to amikacin, ceftazidime, gentamicin, tobramycin and piperacillin. On day 9 of admission, the patient developed fever of 39.5°C, septic shock and multi-organ system failure. Sputum culture grew 2+ C. albicans and 1+ *B. bronchiseptica* again sensitive to the same antibiotics tested previously. Blood culture grew *S. epidermidis*. She expired the following day.

### Case Two

A 51-yr old male presented to the emergency room with headache and confusion. CT of the head revealed four discrete intracerebral tumors consistent with brain metastases. Biopsy of the brain revealed adenocarcinoma of unknown origin. CT of the chest and abdomen and bone scan showed no abnormality. He had a 60 pack-year tobacco smoking history. He was treated with concomitant radiation and chemotherapy. One week after completing a third course of chemoradiation, the patient complained of left groin pain and swelling for 3 days, recurrent fevers with chills, productive cough, confusion, dysuria and multiple skin excoriations in the gluteal area. CT scan of abdomen and pelvis showed incarcerated left scrotal hernia with perforation and retroperitoneal abscess. Chest x-ray was consistent with emphysema and consolidation in bilateral lower lung fields. Blood and urine culture showed no growth. He underwent exploratory laparotomy for incarcerated hernia with resection of sigmoid diverticulitis and drainage of a retroperitoneal abscess with loop colostomy. He was treated with intravenous ampicillin 2gm four times daily, gentamicin 160mg thrice daily, and metronidazole 500mg thrice daily. Two days after admission, chest x-ray showed increased left basilar infiltrates. Blood and urine cultures were negative. Culture from the perineum abscess grew *Bacteroides fragilis*, *Clostridium perfringens*, *viridans Streptococcus*, *E. coli*, and *Enterococcus* spp. Intravenous ampicillin/sulbactam 3g four times daily and fluconazole 200 mg once daily were added, and ampicillin was discontinued. Five days after admission, the patient became increasingly confused and combative. Chest x-ray showed diffuse bilateral infiltrates. Blood culture and urine culture were negative. Sputum cultures grew Candida; Aspergillus flavus and 2+ *Bordetella bronchiseptica* sensitive to cephalothin, ceftazidime, mezlocillin, amikacin, gentamicin, tobramycin, and piperacillin. Fluconazole was discontinued, and intravenous amphotericin B lipid complex 3mg/kg daily was added. On day 8 of hospitalization, CT of the chest revealed diffuse pulmonary interstitial infiltrates and consolidation at the left lung base. After a prolonged hospitalization, on day 26 his condition deteriorated and he expired.

### Case Three

A 54-yr-old moderately obese male presented for a routine follow up chest x-ray. Two years prior, he had undergone surgery followed by chemoradiation for right lung and supraglottal cancer with no evidence of recurrence. The chest x-ray showed a new left lower lobe consolidation with a right pleural effusion. CT of the chest confirmed the presence of the consolidation and was suspicious for malignancy. He underwent bronchoscopy with video-assisted thoracoscopic surgery with wedge resection of the lesion. Histopathology of the tissue demonstrated multiple necrotizing granulomas negative for acid fast bacilli and fungus. Tissue culture grew *B. bronchiseptica*. On admission, he was hoarse with a chronic cough. He had a tobacco smoking history of 70 pack-years. There was no fever, shortness of breath, or chest pain and breath sounds were clear. He remained stable and was discharged five days after surgery. The treatment history was unavailable.

## Discussion

*Bordetella bronchiseptica* infections mainly occur in immunocompromised patients and can cause a variety of respiratory symptoms, ranging from severe to asymptomatic.^1^,^2^ In a review of the literature, the majority of patients infected with *B. bronchiseptica* had at least one predisposing disease such as acute lymphocytic leukemia,^3^ chronic lymphocytic leukemia,^4^,^5^ lymphopenia associated with temolozolide treatment for glioblastoma,^6^ cystic fibrosis^7^ or had undergone hematopoietic stem cell transplantation (HSCT),^8^,^9^ or lung transplantation.^10^ Our study adds three new cases of *B. bronchiseptica* pneumonia to the current literature, which includes a patient with malignant thymoma, a patient with adenocarcinoma of unknown primary origin with brain metastases, and a patient with a history of lung and supraglottic cancer.

It has been suggested that other microorganisms and pathogens often accompany *B. bronchiseptica* pneumonia such as Aspergillus fumigatus, *Klebsiella pneumonia*, *Stenotrophomonas maltophilia*, *Mycobacterium tuberculosis*, *Staphylococcus aureus*, Rhodococcus equi.^9^–^13^ In two of our patients, Aspergillus flavus and Candida albicans were isolated along with *B. bronchiseptica*.

Some common pulmonary infections rather have a similar presentation to *B. bronchiseptica* including *Streptococcus pneumoniae*, *Haemophilus influenzae*, *Mycoplasma pneumoniae* and *Chlamydia pneumonia*. Initial misdiagnoses of *B. bronchiseptica* have included tuberculosis, pneumocystis,^13^ Legionella^14^ and brucellosis.^15^ It is important to consider *B. bronchiseptica* infections when symptoms of *B. pertussis* and *B. parapertussis* are displayed. Patients infected with *B. bronchiseptica* typically present with classic symptoms of pneumonia and in some cases, present with acute sinusitis and bronchitis. They may also exhibit a non-productive “whooping cough” which is also characteristic of B. pertussis, leading to misdiagnosis.^16^

*B. bronchiseptica* and *B. pertussis* both possess the gene for the pertussis toxin but the toxin is only expressed in *B. pertussis*. Alterations in the promoter region of the *ptx* operon in *B. bronchiseptica* lead to transcriptional silencing of the pertussis toxin gene although the gene is biologically active. The *ptx* genes of *B. bronchiseptica* have a different DNA sequence than that of *B. pertussis* and lacks expression due to mutations in the promoter regions.^17^

The current literature does not suggest that cigarette smoking is a risk factor for *B. bronchiseptica* pneumonia. However, each of our three patients reported a history of smoking for 20, 60 and 70 pack years, respectively. One of our patients was diagnosed with emphysema, and a second was being followed up for a history of lung and supraglottic cancer. Shimoni^14^ also reported a case of fatal *B. bronchiseptica* pneumonia in lung cancer patient who had smoked for more than 30 years. The appearance of infections in patients with a mild bronchiectasis, cystic fibrosis and emphysema suggests that the lung diseases, especially those that lead to structural changes, may predispose patients to *B. bronchiseptica* infection.^19^

Virulence factors promoting colonization of *B. bronchiseptica* in animals include filamentous hemagglutinin, fimbriae, and pertactin which help the organisms adhere to the cilia of the respiratory epithelial cells resulting in stasis and difficulty of clearing mucous. In addition, production of adenylate cyclase toxin may interfere with the host immune response.^21^–^25^

In many cases, the origin of the zoonotic infection in immunocompromised hosts is usually through animal contact. Common patient histories include recent contact with ill cats and dogs, healthy dogs,^24^ and contact with newly vaccinated dogs.^27^ Therefore, a history of contact with animals is very important in immunocompromised individuals and such patients should be counseled on how to minimize zoonotic infections. They should be strongly cautioned to seek veterinary consultation for treatment and vaccination of sick pets and to minimize contact with animals when they are ill. Nosocomial transmission of *B. bronchiseptica* has also been reported in the literature.^28^ This suggests that the animal contact is not the only sole route of transmission of *B. bronchiseptica* in immunocompromised patients. Therefore, physicians should be aware of the potential of immunocompromised patients acquiring *B. bronchiseptica* in a healthcare setting.

Previous reports suggest that this organism also exists as a human commensal.^29^–^31^ Diagnosis is based on positive cultures or polymerase chain reaction from a patient with a history of exposure to infected animals.^32^ In cases of pneumonia, cultures from the blood or bronchoalveolar lavage colony counts greater than 10^4^ are useful for diagnosis rather than sputum cultures, as it would be difficult to determine whether *B. bronchiseptica* had any role in the infection or if it were just colonizing the airway. Gram stain of this medium straight rod organism should be reviewed carefully. A good quality sputum gram stain indicating a good number of white blood cells and the presence of gram negative coccobacillary organisms increases the likelihood of *B. bronchiseptica* pneumonia. The organism can be cultured in 48 hours on simple nutritive media at 35 °C where it forms small circular colonies. It can be distinguished from other phenotypically similar organisms using biochemical tests. *B.bronchiseptica* is positive for catalase, urease and oxidase activity, citrate utilization, motility, tetrazolium reduction and growth on salmonella-shigella agar. It fails to grow on potassium tellurite agar. The identification can be confirmed with commercially available tests like Rapid NFT, API-ZYM and Corning N/F system.^33^ There are no lab values or radiographic findings specific for B.bronchiseptica. Previous reports in the radiologic literature have described various findings like multifocal cavitary nodules, ground glass opacities, consolidation, bronchiectasis, mosaic attenuation and interstitial pneumonia.^34^

The response to various antimicrobials is similar to that expected of a gram-negative non-fermentative organism, but it is essential to choose one that has good intracellular penetration.^3^,^4^ Though *B. bronchiseptica* is an extracellular organism, recent studies have shown that this organism is able to invade and persist in eukaryotic cells, like phagocytes and even epithelial cells.^35^,^36^ This invasive property is responsible for chronic or recurrent infection in a host. The pervasive disparity between antibiograms and clinical benefit can be because of patient factors, like the severe underlying disease or immunocompromised state, and different properties of *B.bronchiseptica* like the adenylate cyclase penetration into the polymorphonuclear cells and macrophages leading to inhibition of bacteria killing. Likewise, Kadlec et al found that the beta lactamase gene blaoxa-2 conferred ampicillin resistance to porcine *B. bronchiseptica* isolates while low susceptibility to cephalosporins was based on the low membrane permeability of *B. bronchiseptica.*^37^ The various antibiotics that can be used are aminoglycosides, quinolones, anti-pseudomonal penicillins, tetracycline and TMP-SMZ depending on the susceptibility, though in vitro susceptibility does not reflect in many cases clinical response, because of reasons discussed earlier. Some treatment successes have incorporated combinations of erythromycin, ciprofloxacin and rifampin,^33^ and imipenem.^39^

The duration of treatment has not been established in immunocompromised patients. It may extend anywhere between 2 weeks to 6 weeks depending on the immune status of the patient. Severely neutropenic patients and those with GVHD may require 6 weeks of therapy or even more.^40^–^42^ Chronic or recurrent infection, even after the patient is not in contact with infected animals, suggest epithelial invasion or persistence of the bacterium in macrophages and these cases require an even longer duration of therapy.^43^

## Conclusion

*B.bronchiseptica*, found commonly in the upper respiratory tract of animals, is also a human commensal in the immunocompetent population. However, it can lead to life-threatening infection in those with underlying debilitation or impaired immunity (like patients with neutropenia, diabetes, malnutrition or transplant patients). *B.bronchiseptica* should be considered, in the differential diagnosis, in immunocompromised patients presenting with respiratory symptoms, especially those with known contact with animals. Nosocomial transmission has also occurred, and physicians should be aware that patients with impaired immunity in the healthcare setting may also acquire an infection with *B. bronchiseptica*. The duration and choice of antibiotic is determined on a case by case basis, but treatment with one that has good intracellular penetration is essential because of the organism’s ability to invade epithelial cells and phagocytes.

## Figures and Tables

**Table 1:** Literature review of *B. Bronchiseptica* infection in Immunocompromised Patients

References	Age (yr)	Sex	Malignancy	Risk factors	Comorbid	Treatment	outcome
**2**	55	M	Leukemia	-	Pneumonia	-	-
	60	M	Leukemia	-	Pneumonia	-	-
**5**	60	M	CLL	Prednisone	Pneumonia	Amikacin cephazolin	cured
**6**	56	M	Glioblastoma	-	Chronic cough	-	-
**8**	53	M	Hodgkin’s	-	Pnemonia	-	expire
**9**	20	F	AML	-	Pneumonia	doxycycline ciprofloxacin	cured
**12**	26	F	Hodgkin’s	Prednisone	Pnemonia	cephazolin	cured
**Our Study**	43	F	Thymoma	-	Pneumonia	piperacillin tobramycin	expired
	51	M	Metastatic brain tumor	-	Pneumonia	gentamicin	expired
	54	M	supraglottic cancer	-	pneumonia	-	cured

CLL indicates chronic lymphocytic leukemia; AML, acute myeloid leukemia
